# Global mapping of freshwater contamination by pesticides and implications for agriculture and water resource protection

**DOI:** 10.1016/j.isci.2025.112861

**Published:** 2025-06-09

**Authors:** Yabi Huang, Zijian Li

**Affiliations:** 1 School of Public Health (Shenzhen), Sun Yat-sen University, Shenzhen, Guangdong 518107, China

**Keywords:** environmental science, pollution, water resources engineering

## Abstract

Pesticide residues in freshwater threaten water sustainability, but the global discrepancies in freshwater pesticide contamination remain uncertain. This study collected pesticide concentration data from various countries around the world and adopted a flexible scoring approach to assess contamination within surface freshwater and groundwater. Results revealed significant disparities in contamination among countries. Africa was identified as a priority for freshwater pesticide control as most countries there showed relatively high scores in both surface freshwater and groundwater. Notably, robust pesticide regulations were associated with reduced surface freshwater pollution, whereas pesticide usage intensity showed no clear effect. Additionally, pesticide residues in freshwater pose challenges to drinking water safety, with over half of the water samples in many African and Asian countries exceeding aldrin and dieldrin limits. These findings provide insight into the pattern of global pesticide freshwater contamination, urging efforts on comprehensive water monitoring networks and joint pesticide management within freshwater.

## Highlights

• A global picture of freshwater pesticide contamination is provided
• African countries generally face significant challenges of freshwater pollution
• Stringent pesticide regulations were associated with reduced pesticide pollution
• Excessive pesticide in drinking water is a common issue globally

## Graphical abstract

**Figure:**
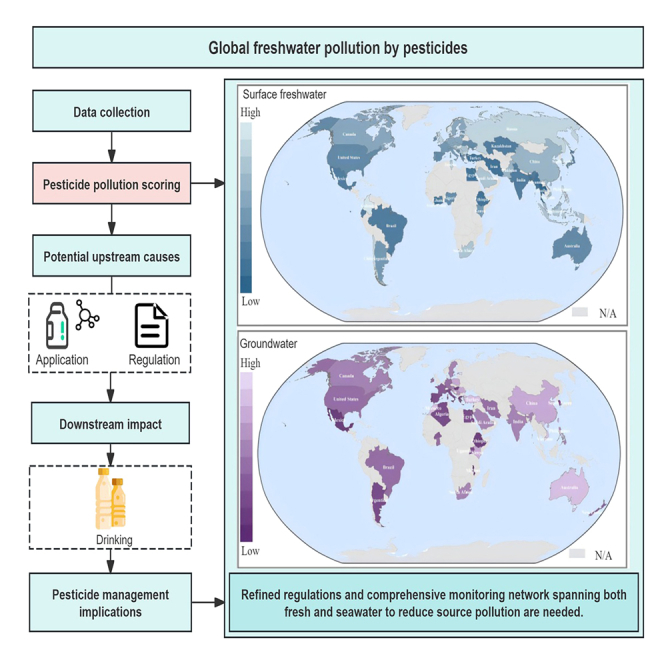

## Introduction

With the global population experiencing dramatic growth, agricultural production becomes increasingly crucial for ensuring food security. Pesticides are extensively utilized worldwide to safeguard crops and plants from pests, insects, and pathogens, thereby ensuring agricultural productivity. However, since pesticides are primarily intended to eliminate specific target organisms, they can inadvertently harm non-target organisms like wildlife and humans.^1^ Following pesticide application, their residues can be transported into various environmental media, including air, soil, surface freshwater, groundwater, biomass, and oceans, potentially impacting environmental quality and heightening health risks for both ecosystems and humans.^2^^,^^3^

Freshwater holds immense significance for human society, particularly as a vital source of drinking water. There are global reports indicating that freshwater, encompassing both surface water bodies and groundwater, has become contaminated with pesticides.^4^^,^^5^ These harmful chemicals can infiltrate surface water bodies like lakes, rivers, and ponds primarily through runoff from agricultural lands, whereas they seep into groundwater through vertical infiltration. Current methods for assessing global pesticide pollution in water include various concentration detection techniques or scoring index systems, such as the Pollution Index (PI)^6^ and the Pesticide Health Risk Index (PHRI).^7^ PI is the ratio of the detected concentration to the water quality standard value. PHRI is computed by comparing three country-specific indicators with the global average level. The calculation of PHRI is dependent on health-based safety levels (e.g., acceptable daily human intake value). In conclusion, these indexes rely on the availability of the corresponding safety thresholds to evaluate the pollution levels. However, for many chemicals, especially emerging compounds, accurate and internationally agreed safety thresholds are still lacking. Zhang and Li^8^ proposed a scoring approach based on the deviation from the central tendency, without referencing safety levels, to investigate and compare the average airborne levels of polycyclic aromatic hydrocarbons in countries around the world. In a previous study,^9^ we successfully applied this method to calculate the deviation of individual countries’ atmospheric pesticide concentrations from the average of all selected countries and then determine the integrated pesticide pollution score for each country. Since this approach is only based on the deviation of environmental concentrations, it is applicable for assessing and comparing water pesticide residual levels across global countries. Pesticide levels in freshwater can be influenced by many factors. Substantial field and modeling studies have illustrated that pesticides commonly used in croplands tend to leach into nearby surface water and groundwater, posing threats to drinking water sources.^10^^,^^11^^,^^12^^,^^13^ Consequently, the use of pesticides in agriculture emerges as a significant upstream factor contributing to potential freshwater contamination. Moreover, environmental regulations, particularly pesticide water quality standards for surface water bodies and groundwater, play a crucial role as downstream factors in preventing freshwater contamination from pesticides. These regulations encompass remediation objectives and regulatory management protocols aimed at safeguarding freshwater ecosystems.^14^ Therefore, investigating both upstream and downstream factors is essential for comprehensively understanding and effectively managing freshwater pesticide contamination.

Considering the extensive use of hundreds of pesticide species and the intricate conditions of freshwater bodies, there have been limited studies evaluating global levels of freshwater pesticide residues, especially regarding upstream and downstream factors such as agricultural practices and regulations. Therefore, the objective of this study is to investigate the global discrepancies in freshwater pollution caused by pesticides and delve into the factors influencing the pollution status and their potential environmental impacts. First, we gathered data on pesticide concentrations in surface freshwater and groundwater from countries worldwide. Subsequently, a scoring approach was employed to calculate the deviation of pesticide concentrations in individual countries from the global average, facilitating the comparison of freshwater pesticide contamination among selected countries. Furthermore, we examined both upstream and downstream factors that could contribute to freshwater pollution by pesticides. Finally, we assessed the potential impacts of freshwater pesticide pollution on drinking water quality. We hope that our study will provide a comprehensive overview of global freshwater contamination caused by pesticides, elucidate potential leading factors, and offer recommendations for pollution management. This study will provide the first comprehensive overview of global disparities in freshwater contamination caused by pesticides, helping to identify priority regions for future pesticide pollution management and promoting future international collaboration for cross-border pesticide contamination.

## Results

### Mapping global surface freshwater contamination

**Figure 1:**
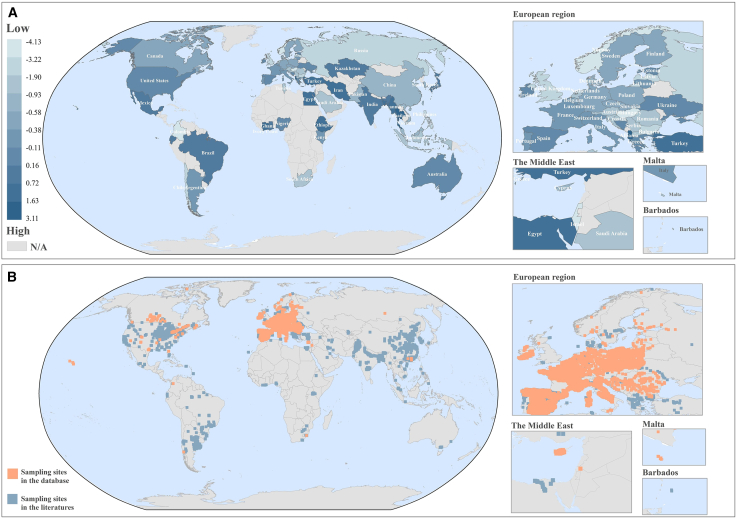
The spatial distribution of pesticide contamination and sampling sites in surface freshwater. (A) Contamination scores. (B) Sampling sites.

To reveal the global occurrence and distribution of pesticides in the surface freshwater, a massive concentration dataset was compiled from 361 studies/databases and the integrated pesticide pollution was analyzed by a scoring approach. Across continents, countries in Africa, South Asia, and Latin America generally received high contamination scores, whereas countries in Europe, had considerably diverse scores (Figure 1A).

Asia and Africa have the largest number of smallholder farms in the world,^15^ yet many of these farmers are poor and lack knowledge and education. They often pursue productivity at the expense of the environment, inevitably leading to pesticide misuse and subsequent surface water pollution.^16^^,^^17^ Countries with scores above 2 included Albania (2.78) and Thailand (3.12), and these scores indicate that concentrations of multiple pesticides in these countries are, on average, approximately two and three orders of magnitude higher than corresponding global values. Health risk assessments were conducted to investigate the potential risks posed by water pesticide contamination in countries with scores exceeding 2. The result revealed that pesticide exposure in surface freshwater posed significant non-carcinogenic risks in these areas (Table S5). For instance, the hazard quotients (HQs) for dicrotophos in Thailand and mirex in Albania reach 458.57 and 8.15, respectively, which are both far greater than 1. Agriculture sectors in these countries contribute significantly to the economy and have employed a large labor force. However, for over a decade, their agricultural sectors have remained undeveloped, with low technological levels and productivity.^18^^,^^19^ The high contamination scores and associated health risks in Albania and Thailand require immediate intervention by local environmental agencies to control pesticide pollution of surface water. In contrast, a total of 22 countries had contamination scores below 0, indicating that the concentrations of pesticides in these countries are generally similar to or lower than global levels. Among these countries, Israel (−4.14), Colombia (−3.71), and Norway (−3.51) obtained the lowest contamination scores. These countries all make efforts to pursue sustainable agriculture with lower chemical pesticide use, such as employing biopesticides,^20^ subsiding sustainable farming practices^21^^,^^22^ and increasing pesticide taxes.^23^ Interestingly, the contamination values in some of the biggest farming countries like the USA, China, and Russia, appear to be less attention-grabbing, warranting further analysis of their agricultural practices and pesticide regulation. Regional-level contamination scores conducted in the USA and China (Figure S1) illustrate the actual pollution disparities among the states/provinces within the countries. This transition in the approach is also applicable to other vast countries like Canada and Brazil. These findings identify regions that urgently need interventions to prevent unsustainable pesticide contamination in surface freshwater.

To evaluate uncertainty in surface freshwater contamination scores, a bootstrap resampling analysis was conducted using China’s surface freshwater data as a case study. The results showed that China’s freshwater pesticide pollution score is −0.59, with a 95% confidence interval (CI) ranging from −0.81 to −0.42 (Figure S2), indicating that the score is statistically robust. Note that the limited or unevenly distributed sampling sites may distort the representativeness of contamination scores in some countries, especially in Africa and Oceania. The representativeness of the contamination scores calculated for each country in this study needs to be interpreted with caution based on the number and distribution of sampling sites. For example, from the data collected, there are only two surface water sampling sites in Australia, so the low contamination scores in Australia (−0.05) may not be representative of actual water pesticide pollution on a national scale. The USA, Canada, and the European region have very robust monitoring infrastructure, including their own comprehensive chemical monitoring systems and databases in surface water (Figure 1B), whereas other vast countries such as Brazil, China, and Russia have not. Given this, our study can provide not only a comparison of contamination levels based on the observed data but also geospatial information and help these countries discover their deficiencies in monitoring sites. Comprehensive and effective surface freshwater monitoring networks are recommended in this study. As monitoring data continue to increase, the extent of contamination at the national scale will become better updated and understood.

### Mapping global groundwater contamination

It is well known that groundwater is the largest available global freshwater resource, so there is also a need to explore pesticide contamination in groundwater worldwide. An extensive dataset of pesticide concentration was compiled from 97 studies/databases, and pollution status was analyzed from a national perspective.

**Figure 2:**
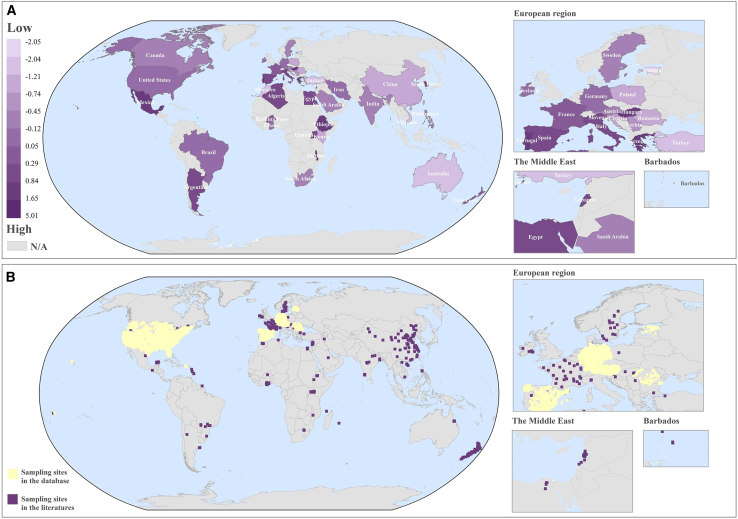
The spatial distribution of pesticide contamination and sampling sites in groundwater. (A) Contamination scores. (B) Sampling sites.

Due to the discrepancies in data collected, the countries with contamination scores in groundwater are not all the same as those in surface water. Slightly different from the pattern of surface water pollution, countries in Africa and the Americas generally appear to have higher scores than in other continents (Figure 2A), indicating noticeable groundwater contamination. In Europe, countries’ scores vary widely, with Greece having a high value (1.36) and Estonia a low (−2.05). On a country scale, Malawi (5.01) in Africa is the only country with a score above 2, and its score indicates that the pesticide concentrations there are, on average, about five orders of magnitude higher than global values. Health risk assessment also identified a significant health threat from cypermethrin exposure in groundwater in Malawi, with HQ values peaking at 57.21 (Table S5). Ethiopia (1.65) and Mexico (1.50) scored behind Malawi. Twenty-four countries received scores below 0, with Estonia (−2.05), Turkey (−1.4), and Vietnam (−1.41) having the lowest values. The global median for groundwater pollution occurred in the United States at −0.08. Regional groundwater contamination scores for the United States and China are displayed in Figure S1. A bootstrap resampling analysis was performed using China’s groundwater data. The computed groundwater pesticide pollution score for China was −0.74, with a 95% CI ranging from −1.21 to −0.58 (Figure S3). It was noteworthy that the extreme scores in some countries, such as Vietnam and Malawi need cautious interpretation owing to the limited sampling sites in this study (Figure 2B). In addition to the extensive or intensive agricultural sectors, a major reason behind groundwater pollution may be the geophysical conditions, such as soil composition, and water table depth, which can affect pesticide penetration.^24^^,^^25^ As seen in the number of sampling sites collected, similar to surface water, long-term groundwater monitoring programs also have been developed in the United States and European region. However, groundwater pesticide monitoring and management is universally neglected in most countries. Combined with contamination scores, these results infer that the contamination risk of usable groundwater now needs to be taken seriously and that a groundwater-level monitoring network should be established to improve assessments of global freshwater resources.

### Correlation between surface freshwater and groundwater contamination

**Figure 3:**
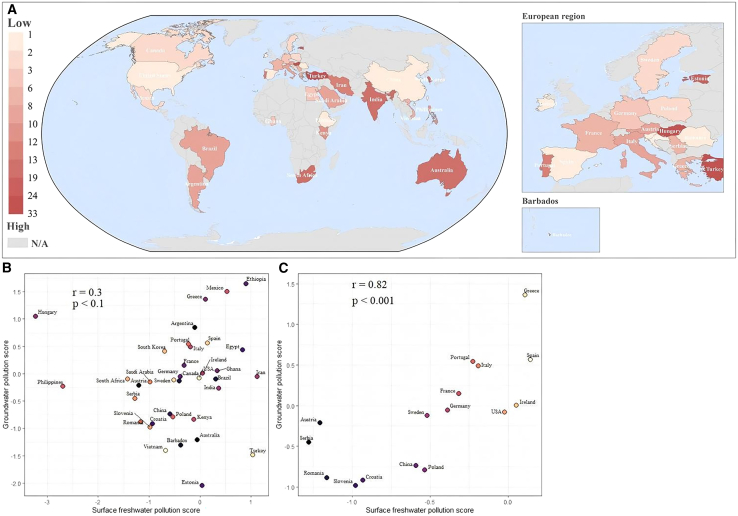
Global disparities between surface freshwater and groundwater pesticide contamination. (A) The global map of rank difference between surface freshwater and groundwater contamination. (B) Scatterplots includes global countries. (C) Scatterplots only includes countries that have sufficient and balanced distributed sampling sites. The p < 0.1 and p < 0.01 labeled in (B) and (C) indicate statistical significance.

One of the sources of groundwater is surface water, and pesticides in surface water may cause contamination of groundwater. To preliminarily explore the potential trend between surface freshwater and groundwater pesticide contamination, this study ranked the countries’ scores of surface freshwater and groundwater separately and mapped the absolute value of the rank difference. The lighter the shade of red of a country, the higher the consistency of pesticide contamination of surface freshwater and groundwater. In addition, the correlation analysis was conducted in freshwater bodies. As shown in Figure 3, the global pesticide residual trends in surface freshwater and groundwater reveal a heterogeneous pattern, and the correlation between their contamination scores is tenuous (r = 0.3, *p* < 0.1). The regression results indicated a weak and statistically insignificant linear relationship (R^2^ = 0.0065, *p* > 0.05). Nevertheless, high consistency is observed in countries with sufficient and balanced distributed sampling sites, such as the United States, China, and most European countries, indicating a similar trend in surface freshwater and groundwater contamination. Similarly, correlation analysis further confirmed a positive relationship between pesticide contamination scores in surface water and groundwater (r = 0.82, *p* < 0.1). The regression result also showed a statistically significant positive association (R^2^ = 0.54, *p* < 0.05). This finding indicates that large uncertainties are expected to be introduced in the comparison process owing to geographic variation in sampling sites of surface and groundwater. Therefore, this inconsistent global trend should be interpreted cautiously. With climate change and socio-economic development, freshwater pollution and scarcity pose significant challenges to sustainability in countries around the world.^26^^,^^27^ For the United States, China, and most European countries, understanding the potential relationship between freshwater bodies could improve the efficiency of water management strategies that address both surface and groundwater pesticide pollution simultaneously.

## Discussion

### Potential influencing factors of freshwater contamination

Building upon the global contamination status, some potential factors influencing freshwater pesticide contamination, including agricultural practices and regulatory performance, were further analyzed to provide insights into pesticide management.

**Figure 4:**
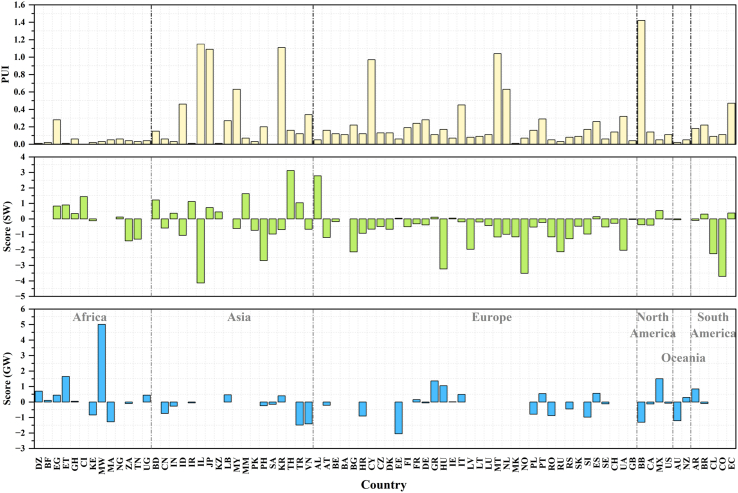
Pesticide usage intensity (t/km^2^) and freshwater contamination scores (unitless) across countries Note: SW and GW mean surface freshwater and groundwater, respectively; missing columns mean no application in the countries.; the countries’ abbreviation list is shown in Table S11.

First of all, pesticide application, one of the agricultural practices, is the major source of pesticide pollution in the environment. Pesticides applied to fields can be transferred to surface freshwater or groundwater by surface runoff or percolation.^25^^,^^28^ Theoretically, the more pesticides applied per unit of agricultural land (i.e., the higher the pesticide use intensity), the greater the transfer to surface freshwater or groundwater, which may lead to more severe pollution levels. In this study, the average pesticide usage intensity (PUI) for each country since 2010 was calculated and compared with the pesticide freshwater contamination scores (Figure 4). Barbados (1.42), Israel (1.15), South Korea (1.11), Japan (1.09), and Malta (1.04) exhibit the highest PUI values. In contrast, Agriculture countries like Brazil (0.22), China (0.06), and the United States (0.1) have considerably lower PUI values. The global pesticide usage intensity and freshwater bodies contamination scores exhibit no clear similar trends, even across continents. For instance, Myanmar with high surface freshwater contamination scores exhibits low pesticide application intensity (0.07). The possible reasons for this inconsistency are manifold. First, the calculation of contamination scores includes both historically and currently used pesticides detected in freshwater, whereas pesticide usage intensity calculation covers only the latter. Some historically used pesticides such as hexachlorocyclohexanes (HCHs), dichlorodiphenyltrichloroethane (DDTs), and endosulfan, have been listed in the Stockholm Convention and prohibited in the 2000s globally due to persistence and toxicity in the environment.^29^ Despite being forbidden, the obsolete stockpiles and large waste volumes of these pesticides still remain legacy issues from the early production, use, and improper disposal.^30^ Second, pesticide residues in freshwater depend not only on the pesticide application amount but also on their leaching proportion. Pesticide fates after application to cropland depend on many factors including its properties, soil conditions, climate, and management practices.^31^^,^^32^^,^^33^ In particular, the physicochemical properties of pesticides such as solubility, volatility, and half-life, which complex interaction with environment compartments. Though Africa’s pesticide usage intensity is relatively low, there are about 190 highly hazardous pesticides registered and in use including persistent acephate, aldrin, and 2,4-D.^34^ Similar situations are occurring in South Asia and Brazil.^35^^,^^36^^,^^37^ As for climate conditions, in humid regions, high precipitation facilitates pesticides to enter into water bodies via surface runoff, resulting in higher pollution levels. Fourth, farmers may use mixtures of pesticides for different pests or diseases, which may result in synergistic effects. In detail, some pesticides may be more persistent in the water than when used alone. This effect may lead to high water pesticide pollution even at low usage intensity. Lastly, countries’ regulation and management of pesticides could also affect pesticide contamination in the freshwater body. In general, countries with stricter pesticide regulations may have lower water pesticide levels.

**Figure 5:**
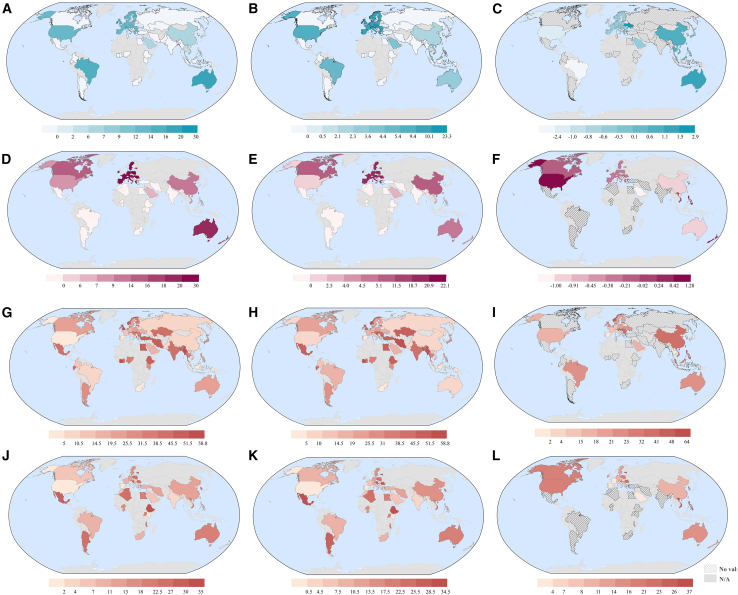
Map of global surface water and groundwater pesticide regulation scores and their rank different with water contamination (A–C and D–F) mean CS, NS_2_, and NS_3_ for surface freshwater and groundwater regulations, respectively. (G–I) The map of rank difference between surface freshwater regulation scores (CS, NS_2_, and NS_3_) and contamination scores. (J–L) The map of rank difference between groundwater regulation scores (CS, NS_2_, and NS_3_) and contamination scores.

Second, once pesticides transfer to surface freshwater or groundwater, their contamination levels may be limited by local pesticide regulations. For instance, countries with stringent pesticide standard values may take certain measures, such as water remediation or biopesticides promotion,^38^^,^^39^ to minimize the residues of chemical pesticides. Therefore, national pesticide regulation is a potential factor influencing pesticide pollution. In this study, completeness (CS) and numeric scores (NS_1–3_) are employed to reflect national regulation performance on coverage and stringency, which may potentially influence freshwater pesticide residues. Among these indicators, NS_1_ is omitted due to non-compliance with calculation prerequisites. As shown in Figures 5A–5F, European countries have the greatest regulation performance in both surface and groundwater, followed by the Oceanian countries, whereas African countries have the worst. For surface water, Ireland (CS = 30; NS_2_ = 22.4; NS_3_ = −0.67) and Switzerland (CS = 30; NS_2_ = 23.3; NS_3_ = −0.65) regulate the most pesticides, and their standards values are also strict. For groundwater, most European countries have the same scores (CS = 30; NS_2_ = 20.8; NS_3_ = −0.20) due to the adoption of uniform standards set by the European Union (EU). Nevertheless, African countries (e.g., Egypt, South Africa, and Ghana) lack standards for all the 30 selected pesticides in both surface and groundwater. These findings point to the regulatory discrepancies of pesticides in freshwater globally and highlight the regions or countries with weak regulatory frameworks.

Figures 5G–5I shows the rank difference for pesticide regulation scores and contamination scores across countries worldwide. The darker color implies a substantial disparity between regulation and contamination. Countries in Africa, West Asia, and Latin America display significant differences between regulation and pollution, which could point to lax regulations, leading to high pollution. Although European countries share similar regulations, they exhibit large variations in the rank difference, suggesting a potential disconnect between regulatory standards and their actual enforcement.^40^ In addition, there are differences in pesticide bans and permission across the EU.^41^ This may affect environmental pesticide pollution. Specifically, even if all EU countries adhere to the same standard values, a country that bans a particular pesticide will likely have a lower water concentration for that substance. Our correlation analysis reveals a statistically significant negative relationship between global surface freshwater contamination scores and both CS and NS2, respectively (r = −0.20, *p* < 0.1; r = −0.25, *p* < 0.1) (Figure S4). The regression result showed statistically significant associations (R^2^ = 0.07, *p* < 0.05; R^2^ = 0.11, *p* < 0.05). Nevertheless, such a statistically significant relationship is not observed in groundwater. This is expected as pesticides in surface water are more directly regulated and managed compared to groundwater. Note that these findings only provide a preliminary and simplified perspective on this relationship. In reality, the relationship between pesticide regulation and contamination in freshwater is multifaceted, as the process from pesticide usage to transfer to freshwater may be influenced by other factors beyond regulation, such as climate and soil conditions.^42^^,^^43^ Further research is needed to untangle these intricate interactions and develop robust regulatory frameworks. A previous study has utilized pesticide bans as an indicator of national pesticide regulation and found that the global risk of pesticide pollution can be reduced by 11% with regulatory convergence (toward the more pesticide-banned side).^44^ In contrast, regulatory scores calculated in this study not only evaluate the correlation between pesticide regulations and water pollution in each country but also identify regulatory deficiencies within individual countries and provide insights for countries to improve their regulatory systems.

Overall, for pesticide practices, inherent discrepancies in the contamination scoring dataset and pesticide application dataset, along with other factors, may distort the potential relationship between pesticide usage intensity and pollution status. Regarding pesticide regulatory performance, statistically significant negative correlations were observed between surface freshwater contamination scores and both CS and NS_2_, highlighting the need for sound pesticide regulation and better implementation of water pesticide policies.

### Impact of freshwater pesticide contamination on drinking water quality

**Figure 6:**
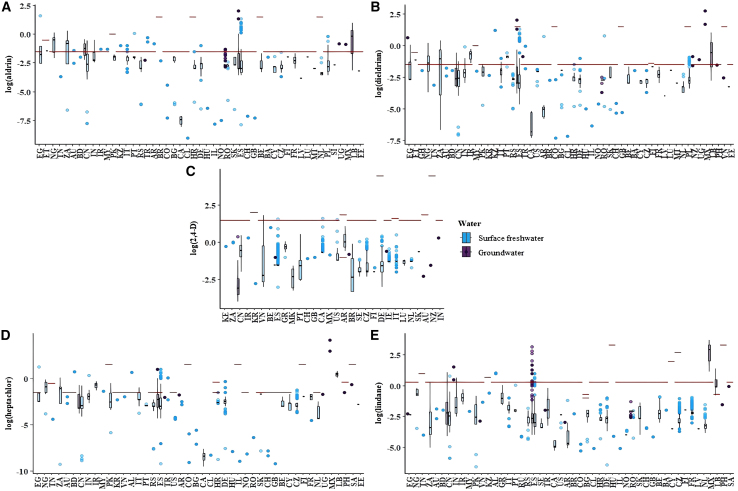
Boxplot of selected pesticide concentration in countries worldwide (A) aldrin, (B) dieldrin, (C) 2,4-D, (D) heptachlor, (E) lindane. Red line denotes the levels of drinking water standards in countries; countries’ abbreviation list is shown in Table S16.

Surface freshwater and groundwater are both key sources of drinking water supplies.^45^ The observed contamination in freshwater can have direct implications for drinking water quality, especially in most developing countries and regions where residents often consume untreated freshwater directly.^46^ In this case, freshwater pesticide pollution can have a great impact on drinking water safety. This study investigates whether pesticide levels exceed drinking water thresholds when freshwater is used directly for drinking purposes. The selected pesticide concentrations for countries worldwide and their corresponding drinking water quality are presented in Figure 6. For global freshwater samples (both surface water and groundwater) detected with heptachlor, aldrin, dieldrin, lindane, and 2,4-D, about 5.9%, 4.8%, 4.6%, 1.3%, and 0.2% exceed the standard set by corresponding countries. In most African and Asia countries, samples have an exceedance rate of up to 50% or more for aldrin and dieldrin, which indicates that untreated freshwater in these areas is not suitable for direct drinking use and the consequent hazards should be paid attention to. Africa has increased the pesticide in the past decade and even registered and imported toxic pesticides (e.g., heptachlor, aldrin, and dieldrin) that have been highly banned by the European Union.^34^ It is reported that possible health risks linked with aldrin and dieldrin include but are not limited to hepatic and reproductive dysfunctions and CNS symptoms.^47^^,^^48^ As for countries in the Americas and Oceania, the number of the detected samples is small, so relatively high exceedance rates (14.3%–50%) of freshwater are found in Mexico, New Zealand, and Argentina. For EU countries, they have to process freshwater for drinking purposes to remove contaminants (Directive EU 2020/2184). According to a report from the United Nations,^49^ most EU countries have less than 1% of their drinking water directly from untreated surface water or groundwater. Therefore, the exceedance of pesticides for water in EU countries is not applicable here. It is noteworthy that the rate of exceedance of these pesticides in freshwater should be compared with caution among countries due to differences in sample number and drinking water standards set in each country.

Overall, in most countries around the world, pesticide residues in freshwater are excessive for drinking purposes. To solve the contemporary concern of water scarcity and to improve clean water, it is imperative to monitor and control freshwater pesticide contamination.

### Recommendations

This study provides a comprehensive picture of the existing observation data of pesticides in surface water and groundwater and explores their correlations and potential impact factors. Our findings indicate that most countries in Africa face significant challenges of freshwater pollution. Meanwhile, surface water and groundwater exhibit similar trends in pesticide pollution within freshwater bodies. These findings highlight the priority areas for pesticide control and the necessity of a joint management strategy within freshwater bodies. Due to other factors like climate and pesticide properties beyond application, the connection between gross pesticide usage intensity and freshwater pollution is unclear. In contrast, variations in surface water pollution levels across countries appear to be associated with differences in pesticide regulations. This analysis suggests several potential strategies to mitigate the pressing issue of freshwater pesticide pollution for high-score regions. At the regulation level, strengthening regulations on pesticides through stricter standards and enforcement may be able to control pesticide pollution to prescribed levels. At the technical level, developing and using high-effect and low-toxic green alternatives to conventional pesticides can reduce the pesticide usage intensity and address the source of pesticide pollution. This study also found that pesticide levels in untreated freshwater frequently exceeded drinking water standards, which should be of concern in areas where freshwater is often used directly for drinking purposes. These findings emphasize the necessity of refining pesticide regulations and establishing a comprehensive monitoring network spanning freshwater to reduce source pollution and future costs of water treatment. The distribution of sampling sites collected in this study provides a reference to optimize monitoring networks, particularly in regions with significant data gaps like Oceania and Africa. It is recommended to utilize this distribution pattern to strategically enhance monitoring site density, which could facilitate more accurate pollution assessments. To enhance global data consistency and quality, establishing international data cooperation alongside the monitoring networks is recommended. This integrated approach would not only improve the comprehensiveness of pesticide data but also enable more robust cross-regional comparisons. It should be noted that although this study could not directly prove a causal relationship between freshwater pesticide contamination and potential impact factors, the trends between them could provide an important basis for future in-depth research.

### Limitations of the study

Some limitations and research gaps are identified in our analyses, prompting suggestions for future studies. First, due to the restraint of available concentration data, uncertainty may be introduced in the scoring process. It should be pointed out that our scoring approach is flexible and can include more data to improve the representativeness of countries’ scores in the future. Meanwhile, the limited sampling sites observed in certain countries also underscore the significance of the water monitoring network. Second, except pesticide usage intensity, other agricultural practices such as crop structure and pesticide type are not taken into account due to the lack of detailed data. That said, future studies can further extend this section to include other components. Third, even after clarifying the pattern and relationship between freshwater pesticide pollution and the oceanic environment, understanding the environmental exchange behavior among these compounds remains elusive. Fourth, since this study focuses on water pesticide concentrations, more research is needed on the effects of water pesticide pollution on long-term ecological or human health. In addition, environmental pesticide fate is complex and could be influenced by multiple factors. Future research can investigate how climate change affects the leaching of pesticides into freshwater bodies and the patterns of water pesticide pollution.

## Resource availability

### Lead contact

Further information and requests for resources should be directed to and will be fulfilled by the lead contact, Zijian Li (lizijian3@mail.sysu.edu.cn).

### Material availability

This study did not generate new unique reagents.

### Data and code availability

• All data in this study are reported in the supplemental information and will be publicly available upon publication.
• This paper does not report custom code.
• Any additional information required to reanalyze the data reported in this paper is available from the lead contact upon request.

## Acknowledgments

This work was financially supported by the 10.13039/501100001809National Natural Science Foundation of China (grant no. 42107495).

## Author contributions

Y.H., writing—review & editing, writing—original draft, methodology, and data curation. Z.L., writing—review & editing, writing—original draft, methodology, funding acquisition, data curation, and conceptualization.

## Declaration of interests

The authors declare no competing interests.

## STAR★Methods

### Key resources table

**Table:** 

REAGENT or RESOURCE	SOURCE	IDENTIFIER
**Software and algorithms**		

R 4.0.2	R Core Team	https://www.r-project.org/
ArcGIS 10.8	Environmental Systems Research Institute	https://www.arcgis.com/index.html; RRID SCR_011081
Microsoft Excel 2021	Microsoft	https://excel.cloud.microsoft/

**Other**		

Any additional information required to reanalyze the data reported in this paper	Available by request from lead contact	N/A

### Method details

#### General method

The overall framework used to investigate the issue of global freshwater pollution caused by pesticides is illustrated in Scheme S1. Initially, we collected data on pesticide levels in surface freshwater and groundwater from various countries around the world. To quantify the pollution levels in freshwater, we employed a scoring method for the selected countries. By utilizing these contamination scores, we delved deeper into the relationship between pollution levels in surface freshwater and groundwater. In order to identify the primary causes of global freshwater pollution due to pesticides, we gathered information on the intensity of pesticide usage across countries worldwide. This data serves as a potential upstream factor leading to freshwater pollution. Lastly, we examined the regulations pertaining to pesticide usage in freshwater bodies, which could serve as a downstream measure to prevent freshwater pollution. Lastly, we assessed the impact of current freshwater pollution levels on drinking water quality This involved comparing the freshwater pesticide levels against established drinking water quality standards.

#### Freshwater pesticide concentration collection

In this study, a thorough literature search to comprehensively was conducted to identify pesticide concentrations in global freshwater. Our search encompassed the Web of Science database and official environmental monitoring program websites. To ensure a comprehensive search, we used the following keyword groups with AND-operators: "freshwater" or "river" or "lake" or "groundwater" or "well", "pesticide" or "insecticide" or "herbicide" or "fungicide", and "residuals" or "pollution" or "concentration". We focused on publications from 2010 to 2023.

Additionally, we included papers that were not found through the database search but were referenced in other studies. To determine inclusion criteria, we considered studies that met the following conditions: (ⅰ) written in English, (ⅱ) published in peer-reviewed journals, (ⅲ) investigated pesticide concentrations in surface freshwater or groundwater (e.g., rivers, lakes, wells), (ⅳ) included sampling dates from 2010 onwards, (ⅴ) provided specific pesticide concentration data (not just pictures), and (ⅵ) offered necessary sampling information such as location and year. We excluded literature reviews and meta-analyses from our analysis. Furthermore, articles that proposed pesticide concentration predictions in freshwater based on previous research or used national monitoring data for analysis were also not considered. Following these criteria, a total of 361 surface freshwater studies/databases from 71 countries and 97 groundwater studies/databases from 43 countries were selected (Tables S1 and S2). Pesticide concentrations were extracted from these articles, and raw official monitoring databases were processed following the protocol by Wolfram^50^ (Method S1). To ensure consistency, all concentration units were converted to μg/L. Moreover, each pesticide was assigned a unique Chemical Abstract Service Registration Number (CAS No.) based on the PubChem database. Information about the temporal distribution of the collected data within the 2010–2023 period is displayed in Figures S5 and S6.

#### Global freshwater contamination scores

To compare the extent of freshwater contamination by multiple pesticides among chosen countries, we employed a scoring method to assign a score to each country.^8^ This scoring method was devised by calculating the deviation of freshwater pesticide concentrations in each country from the overall central tendency among all selected countries. High scores indicate that pesticide concentrations in a country are generally higher than the corresponding global average. The mathematical formula for this approach is as follows:

(Equation 1) $$S=\underset{Sites}{\underset{︸}{\frac{1}{M}\sum_{m=1}^{M}\left\{\overset{Pesticides}{\overset{︷}{\frac{1}{N}\sum_{n=1}^{N}\left[\mathrm{Log}\left[C_{n,m}\times I\left(C_{n,m}\right)+LOD\times I\left(LOD\right)\right]-\overset{Central\;tendency}{\overset{︷}{\bar{{LogC}_{n}}}}\right]}}\right\}}}\forall \;M\geq 1,N\geq 1$$

(Equation 2) $$I\left(C_{n,m}\right)=\left\{ \begin{array}{c} 1;\;if\;detectale\\ 0;\;if\;undetectable \end{array}\right.;\;I\left(LOD\right)=\left\{ \begin{array}{c} 0;\;if\;detectale\\ 1;\;if\;undetectable \end{array}\right.$$

Where S is the dimensionless contamination score of freshwater pesticides in the specific country and its value quantifies how much the pesticide concentrations in a specific country deviate from the global average: S ≤ 0 signifies the levels of pesticides are generally similar to or below the global average, 0 < S < 2 suggests slightly higher levels and S ≥ 2 means significantly higher pesticide levels. It should be noted that the threshold of S is flexible and can be adjusted depending on the specific focus of the future study. $\mathrm{Log}\left[C_{n,m}\times I\left(C_{n,m}\right)+LOD\times I\left(LOD\right)\right]$ denotes the log (10)-transformed value of pesticide (n) concentration in the sampling sites (m) of the country. Mean concentrations extracted from the literature are used to indicate pesticide concentration $C_{n,m}$. $I\left(C_{n,m}\right)$ and I(LOD) are indicator functions calculated by Equation 2. LOD means the limit of detection and when it is not available, the limit of quantification (LOQ) can also be used for the calculation. $\bar{{LogC}_{n}}$ represents the global central tendency of logarithmic concentration of pesticide n (${LogC}_{n}$), calculated as the site-weighted means of ${LogC}_{n}$ across all the selected countries. The formula is as follows:

(Equation 3) $$\bar{{LogC}_{n}}=\frac{1}{Q}\sum_{q=1}^{Q}{LogC}_{n}$$

where Q represents the overall number of sampling sites worldwide. $C_{n}$ refers to the concentration of pesticide n in the specific country. The involved parameters (e.g., pesticide n and sampling site m) and detailed calculations for surface freshwater and groundwater are displayed in Tables S3 and S4, respectively. Note that, the contamination score serves as a relative indicator that is applied to compare integrated pesticide residual levels between countries. It does not involve any safety thresholds and thus does not directly represent the severity of pollution risk. To further investigate whether high relative pesticide contamination levels may cause potential health hazards, health risk assessments are conducted only for countries with scores exceeding 2, using the USEPA model^51^ (see Method S2). The calculation process is shown in Table S5.

Mixture concentrations (e.g., ∑DDTs and ∑HCHs) are excluded from the calculation due to the unclear proportion of their components. Note that, for a country, the score value produced by the aforementioned approach will better represent the actual relative pollution status when the input data are sufficient and geographically balanced. Furthermore, this flexible approach also allows for transitions from a national scale to a regional scale, to analyze internal pollution status within some expansive countries such as the USA and China (Tables S6, S7, S8, and S9). To preliminarily explore the potential trend between surface freshwater and groundwater pesticide pollution, countries’ scores are sorted from high to low in each water body and then the absolute value of the rank difference is mapped (Table S10). The lower the absolute value, the higher the consistency in the pollution trend between the two water bodies.

#### Data quality and uncertainties

To address potential uncertainties stemming from data sources and calculation methodology, several measures were taken to enhance the robustness of data analysis in this study. Firstly, although sampling campaigns were unevenly distributed across years and countries, such temporal variability was mitigated by restricting data to 2010–2023 and averaging concentrations at the sampling site level. Second, to ensure spatial representativeness, pollution scores were sampling site-weighted, and the spatial distribution of sampling sites was mapped to facilitate interpretation. Thirdly, given that the data were non-normally distributed and had extreme values, logarithmic transformation, and Spearman correlation were applied in the analysis to ensure statistical robustness. To estimate the impact of data uncertainties on contamination scores, the bootstrap resampling method was supplemented. Specifically, given that China had the highest number of relevant literature records in our study, we conducted 1000 resampling iterations using Chinese data from Tables S3 and S4 as examples to estimate the variability in the pollution scores and derive 95% confidence intervals (CI), which can provide a statistically sound inference range.

#### Pesticide use intensity

In order to investigate potential upstream factors contributing to freshwater pesticide contamination, we gathered data on pesticide usage and agricultural land area for selected countries from the FAO database (updated to 2021).^52^ Subsequently, we calculated the average pesticide usage intensity (PUI, t/km^2^ per year) for each country from 2010 to 2021, using the following equation:

(Equation 4) $$\bar{PUI}=\frac{1}{12}\sum_{i=2010}^{12}\frac{{PU}_{i}}{{AL}_{i}}$$

Where ${PU}_{i}$ and ${AL}_{i}$ denotes the pesticide usage amount (t) and agricultural land area (km^2^) for a country in the year i. Detailed data and calculations are shown in Table S11.

#### Global freshwater regulation

To delve into potential upstream factors contributing to freshwater pesticide contamination, we gathered pertinent water quality standards (WQS) concerning surface freshwater and groundwater, for the selected countries from existing regulatory studies.^14^^,^^53^ Additionally, the influence of pesticide regulation on freshwater pesticide residues is assessed by assigning regulation scores for each selected country.

The regulation scores were devised based on pesticide water quality standards, encompassing both completeness and numerical aspects.^54^ The former gauges whether a country regulates a sufficient number of pesticides in surface freshwater or groundwater, while the latter evaluates whether the WQS set by a country is stringent enough to safeguard freshwater from pesticide pollution. In this study, we focused on the 30 most commonly detected pesticides in freshwater bodies to construct the regulatory score (Tables S12 and S13). The mathematical formulas for defining the pesticide regulation score are expressed as follows:

(Equation 5) $${CS}_{j}=\sum_{i=1}^{30}{WQS}_{i,j};\;{\forall WQS}_{i,j}=\left(1;\;if\;there\;is\;a\;water\;quality\;standard\;for\;pesticide\;i;0,if\;not\right)$$

(Equation 6) $${NS}_{1,j}=\sum_{i=1}^{30}\left\{1.0-Normdist\left[\frac{\log_{10}\left({WQS}_{i,j}\right)-\mu_{Li}}{\sigma_{Li}}\right]\right\}$$

(Equation 7) $${NS}_{2,j}=\sum_{i=1}^{30}\left\{1.0-\frac{\log_{10}\left({WQS}_{i,j}\right)-\mathrm{Min}\left[\log_{10}\left({WQS}_{i}\right)\right]}{\mathrm{Max}\left[\log_{10}\left({WQS}_{i}\right)\right]-\mathrm{Min}\left[\log_{10}\left({WQS}_{i}\right)\right]}\right\}$$

(Equation 8) $${NS}_{3,j}=\sum_{i=1}^{N}\left\{\left[\frac{\log_{10}\left({WQS}_{i,j}\right)-\mu_{Li}}{N}\right]\right\}$$

${CS}_{j}$ denotes the completeness score in country j and is computed based on the regulated number of 30 selected pesticides for freshwater. Higher CS values indicate more comprehensive pesticide regulation. ${NS}_{1,j}$ , ${NS}_{2,j}$ and ${NS}_{3,j}$ represent numerical scores for the country j obtained by three computational methods. In detail, ${NS}_{1,j}$ is calculated as the total probability that a random standard exceeds the country’s WQS based on the log-normal random distribution. ${NS}_{2}$ quantifies the relative position of the WQS between the extreme values in the distribution. ${NS}_{3}$ is calculated based on the average deviation of WQS to the global central tendency. Higher values of ${NS}_{1}$ and ${NS}_{2}$, as well as a lower value of ${NS}_{3,j}$, indicate more conservative pesticide regulations in a country.

In the Equations 6, 7, and 8, $\log_{10}\left({WQS}_{i,j}\right)$ is the logarithmic standard value for pesticide i in country j. $\log_{10}\left({WQS}_{i}\right)$ denotes the set of global logarithmic standard values for pesticide i. $\mu_{Li}$ and $\sigma_{Li}$ are the mean and standard deviation of $\log_{10}\left({WQS}_{i}\right)$, respectively. Assuming that WQSs conform to log-normal random distributions, Normdist is the function to calculate the cumulative probability of $\log_{10}\left({WQS}_{i,j}\right)$. Min and Max are the function to determine the extreme values for $\log_{10}\left({WQS}_{i}\right)$. N represents the total number of pesticides regulated in country j. Detailed parameters and relevant computations are shown in Tables S12 and S13.

Noting that if a nation has developed more than one pesticide standards system in freshwater, only the most conservative one is included in the analysis. The Shapiro-Wilk normality test found the non-normality of WQSs (*p* < 0.05), therefore NS_1_ has not discussed discussion due to the violation of the prerequisite assumption. The potential trend between regulation and contamination scores was also mapped by the rank difference (Table S14).

#### Global drinking water regulation

Generally, surface freshwater and groundwater serve as the two main sources of drinking water. According to the report from the United Nations,^49^ the proportion of the global population using safely managed drinking-water services is only 69%, and the proportion of the population directly using surface freshwater or groundwater as drinking water is up to 25% in Nigeria (Table S15). To ascertain whether global freshwater pollution by pesticides will impact the quality of drinking water, we compared the gathered freshwater pesticide concentrations with the respective national-level drinking water quality standards in each country. These standards were obtained from existing regulatory studies^55^ (Table S16). For our analysis, we focused on five commonly detected and regulated pesticides, that is, Aldrin (CAS 309-00-2), Dieldrin (60-57-1), 2,4-D (94-75-7), Heptachlor (76-44-8) and Lindane (58-89-9). In cases where standard values were absent in a country, standards set by the World Health Organization were applied.

### Quantification and statistical analysis

The Shapiro-Wilk test was used to assess normality, and the results indicated that the data were not normally distributed (*p* < 0.05). Accordingly, Spearman’s rank correlation was performed for correlation analysis, with statistical significance set at *p* < 0.1. Additionally, simple linear regression analysis was supplemented to avoid potential spurious correlations, treating surface water contamination scores as the independent variable and groundwater scores as the dependent variable. Data management and analysis were completed using Microsoft Excel and R software. The data visualization was conducted using ArcGIS. R programming language includes ggplot and ggpubr.

## Supplemental information

Document S1. Figures S1–S6, Scheme S1 and Methods S1, S2

Table S1. Pesticide concentrations in surface freshwater

Table S2. Pesticide concentrations in groundwater from literature

Table S3. Calculation of pesticide pollution scores in surface freshwater

Table S4. Calculation of pesticide pollution scores in groundwater

Table S5. Health risk assessments for countries with pollution scores above 2

Table S6. Calculation of pesticide pollution scores in surface freshwater by China

Table S7. Calculation of pesticide pollution scores in surface freshwater by USA

Table S8. Calculation of pesticide pollution scores in groundwater by China

Table S9. Calculation of pesticide pollution scores in groundwater by USA

Table S10. Rank difference of surface freshwater and groundwater pollution among countries

Table S11. Calculation of pesticide usage intensity

Table S12. Calculation of regulation scores (CS, NS1–3) for surface freshwater

Table S13. Calculation of regulation scores (CS, NS1–3) for groundwater

Table S14. Rank difference of surface freshwater and groundwater regulations among countries

Table S15. Drinking water estimates by country

Table S16. Worldwide drinking water standards for selected countries
