# Supplementary material for: Global mapping of freshwater contamination by pesticides and implications for agriculture and water resource protection
Source: iScience. 2025 Jun 9;28(7):112861. doi: 10.1016/j.isci.2025.112861 (PMC12256343; doi:10.1016/j.isci.2025.112861)
Supplement: Document S1. Figures S1–S6, Scheme S1 and Methods S1, S2 [file mmc1.pdf]

iScience, Volume *28*

Supplemental information

Global mapping of freshwater contamination by pesticides and implications for agriculture and water resource protection

Yabi Huang and Zijian Li

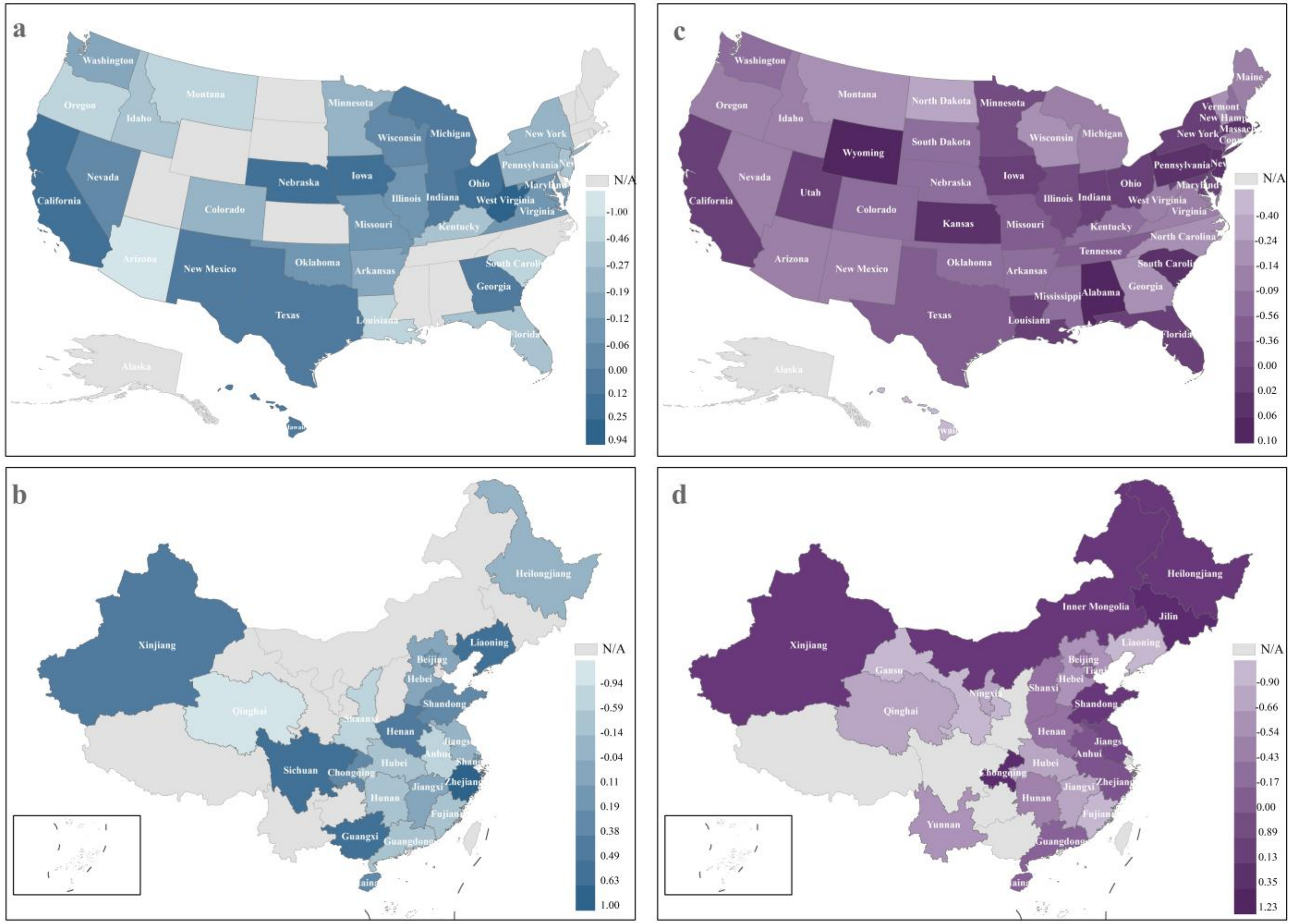

Figure S1. The spatial distribution for freshwater in the USA and China (a and b for surface freshwater; c and d for groundwater), related to the Results (“Mapping global surface freshwater contamination” and “Mapping global groundwater contamination”).

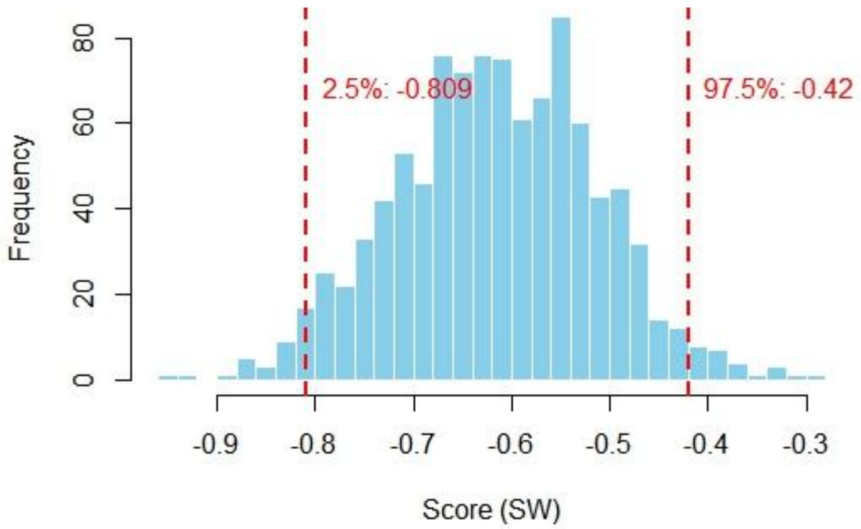

Figure S2. Bootstrap distribution of Score (SW) in China, related to the Results ("Mapping global surface freshwater contamination").

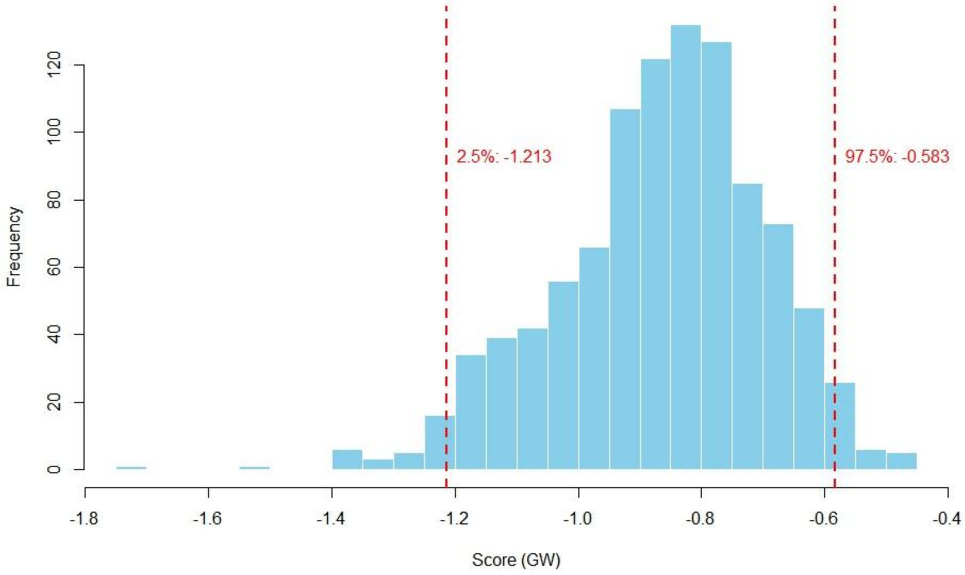

Figure S3. Bootstrap distribution of Score (GW) in China, related to the Results ("Mapping global groundwater contamination").

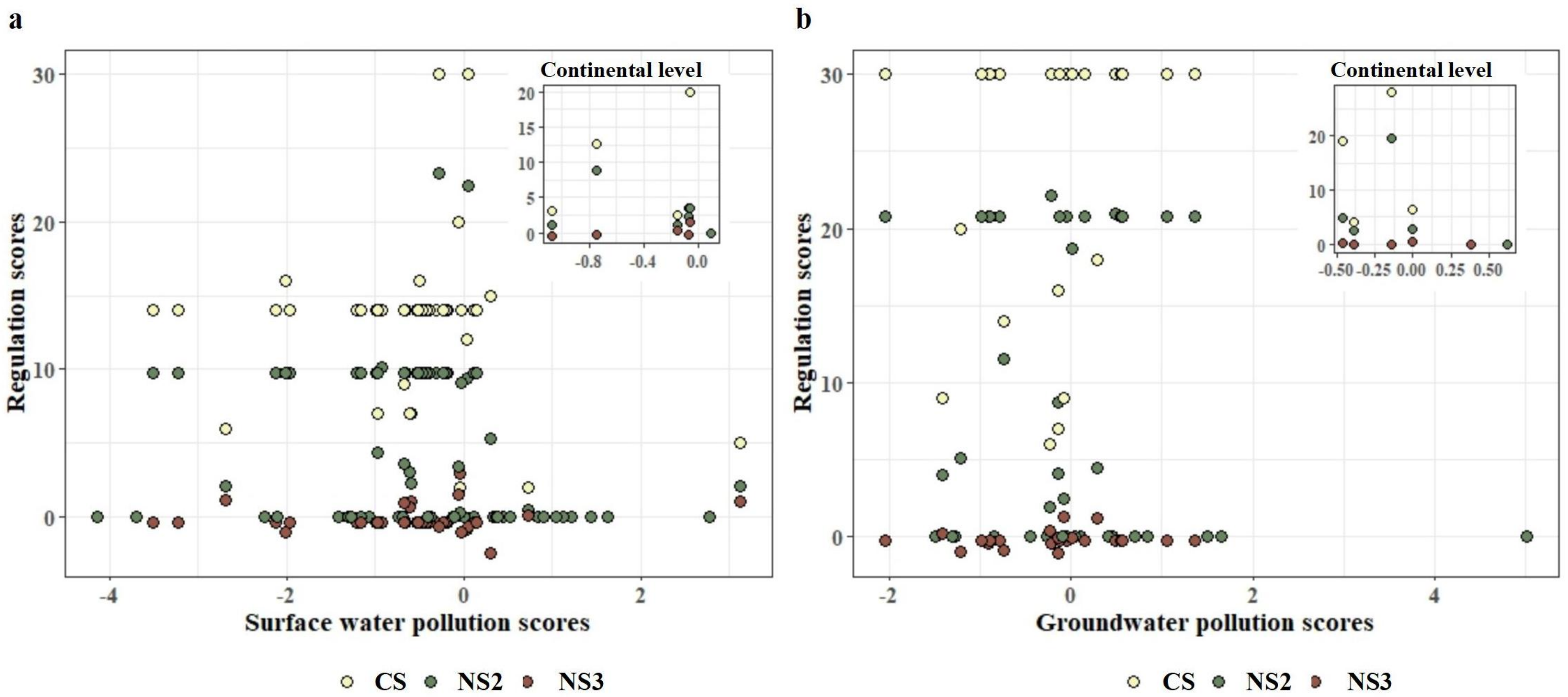

Figure S4. Scatter plot of regulation scores and freshwater pollution scores (a: surface freshwater; b: groundwater), related to the Discussion ("Potential influencing factors of freshwater contamination").

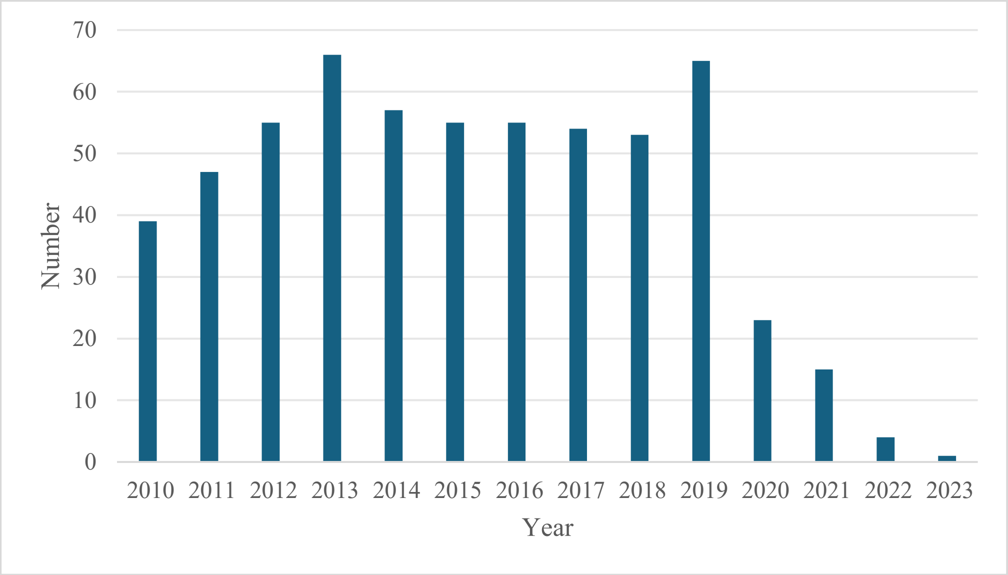

Figure S5. Temporal distribution of the surface water data, related to the STAR Methods (“Freshwater pesticide concentration collection”).

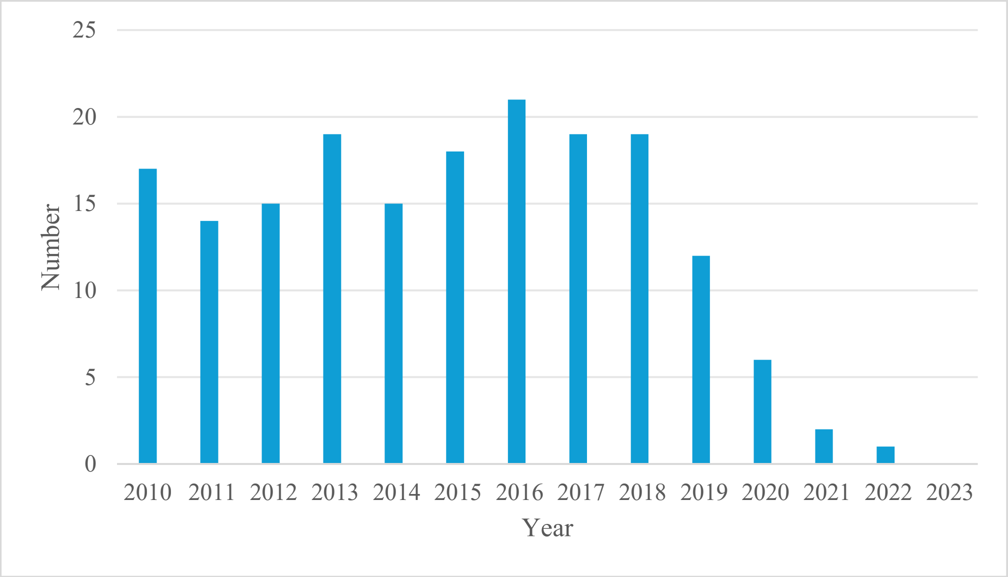

Figure S6. Temporal distribution of the groundwater data, related to the STAR Methods ("Freshwater pesticide concentration collection").

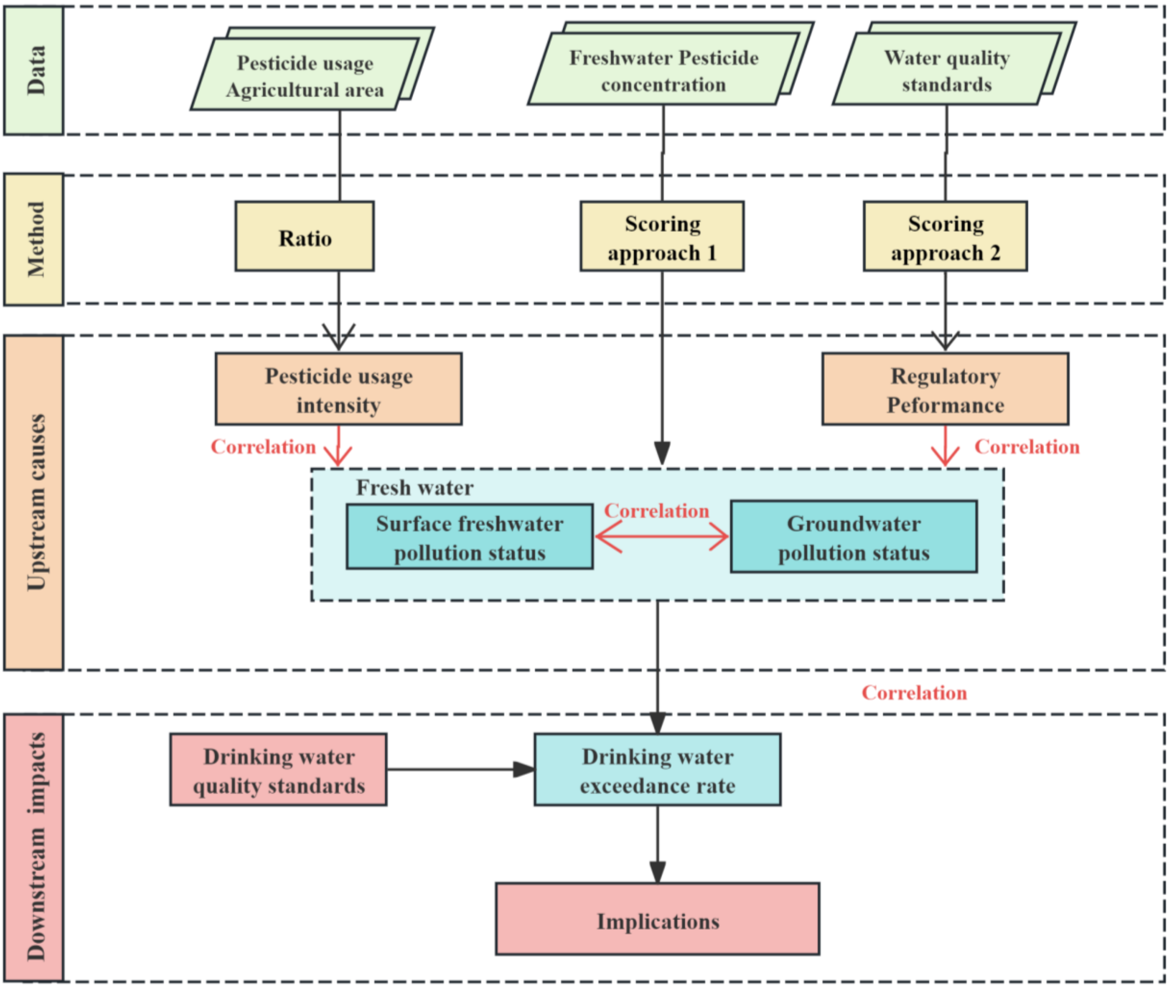

Scheme S1. Flow chart of the methodology in this study, related to the STAR Methods ("General method")

Supplemental Method

Method S1: Database processing

In summary, the first step involved extracting all measurements taken after 2010, while removing non-freshwater data (such as soil and air). Furthermore, if a database differentiated between surface water suspended sediment and dissolved phase concentrations, only the latter were retained. Next, the detection data pertaining to non-pesticide compounds was eliminated by cross-referencing the compounds with the PPDB, PAN, and PubChem websites to confirm their classification as pesticides. In addition, remove those data identified as duplicates in location/ID× time × pesticide dimensions, as well as those illogical data (e.g., minimum>maximum). Finally, concentrations from the same monitoring site, sampled year, and pesticide compound were compiled to derive the annual maximum, median, and mean concentrations.

Method S2: Human health risk assessment

To further investigate the actual health hazard posed by pesticide contamination in countries with a score greater than 2, we conducted non-carcinogenic health risk assessments using the following equations[1]:

$$ADD = \frac{C_{water} \times IR \times EF \times ED}{BW \times AT} \times 10^{-3} \quad \text{(s1)}$$

$$HQ = \frac{ADD}{RfD} \quad \text{(s2)}$$

Where ADD (mg/kg/day) means the average daily intake dose of the pesticide via drinking water. C_{water} (μg/L) is the pesticide concentration collected in a specific country. IR (2 L/day) and BW (60 kg) are the daily water ingestion rate and body weight. of a healthy adult[2,3]. EF (365 days/year) and ED (70 years) are exposure frequency and duration. AT (25550 days) is the average exposure time[4]. 10^{-3} is the unit conversion factor. HQ (unitless) is the hazard quotient and HQ value >1 indicates a significant health risk. RfD (mg/kg/day) is the intake reference dose obtained from official databases[5,6]. The detailed parameters and calculation process are shown in Supplementary Table S5.

Supplemental Reference

1. USEPA (2023). Regional Screening Levels (RSLs)—User's Guide (November 2023). https://www.epa.gov/iris.

2. WHO (2017). WHO World Health Organization Guidelines for Drinking-Water Quality: Fourth Edition Incorporating the First Addendum. Geneva: Licence: CC BY-NC-SA 3.0 IGO. http://apps.who.int/iris.

3. El-Nahhal, I., and El-Nahhal, Y. (2021). Pesticide residues in drinking water, their potential risk to human health and removal options. J Environ Manage *299*, 113611. https://doi.org/10.1016/j.jenvman.2021.113611.

4. Kruć-Fijałkowska, R., Dragon, K., Drożdżyński, D., and Górski, J. (2022). Seasonal variation of pesticides in surface water and drinking water wells in the annual cycle in western Poland, and potential health risk assessment. Sci Rep *12*, 3317. https://doi.org/10.1038/s41598-022-07385-z.

5. EPA (2011). Integrated Risk Information System. Integrated Risk Information System. https://www.epa.gov/iris.

6. Lewis, K., Tzilivakis, J., Green, A., and Warner, D. (2006). Pesticide Properties DataBase (PPDB). University of Hertfordshire. https://sitem.herts.ac.uk/aeru/ppdb/en/index.htm.
